# The relationship between early childhood development and feeding practices during the dietary transitional period in rural China: a cross-sectional study

**DOI:** 10.3389/fpubh.2023.1202712

**Published:** 2023-08-11

**Authors:** Yihua Liu, Chun Chang

**Affiliations:** Department of Social Medicine and Health Education, School of Public Health, Peking University, Beijing, China

**Keywords:** early childhood development, feeding practices, dietary transitional period, rural China, ASQ-C

## Abstract

**Introduction:**

Early childhood feeding environments and practices not only affect children's nutritional status but also provide children with a variety of external stimulations to affect the development of the child's brain, especially for the first 1,000 days of children. The relationship between early childhood development (ECD) and feeding practices during the dietary transitional period has not previously been described.

**Method:**

This study used quantitative survey data from the Integrated Early Childhood Development Project to investigate this association between ECD and feeding practices during the dietary transitional period in poor rural areas of China. Data concerning the child and family characteristics and feeding practices were collected through the questionnaire completed by caregivers. Developmental delays were explored through a five-pronged, structured, parent-completed Age and Stage Questionnaire. The chi-squared test and multivariate logistic regression analyses were used to explore the associated factors in ECD.

**Results:**

The results showed that 33.6% of children had at least one area of developmental delay during the dietary transitional period. Of all five regions evaluated, the prevalence of fine motor developmental delays was highest (17.7%), followed by communication (14.9%), problem-solving (13.8%), personal-social skills (11.9%), and gross motor (11.8%), respectively. Significant predictors of increased odds of developmental delay included types of complementary foods (OR = 0.70, 95% CI = 0.53–0.94), adequate feeding frequency (OR = 0.69, 95% CI = 0.52–0.90), and breastfeeding time and bottle feeding (OR = 0.66, 95% CI = 0.50–0.88).

**Discussion:**

According to the results, a high prevalence of developmental delay was observed in children during the dietary transitional period in the rural areas of China. The feeding practices of children were associated with their developmental status, including factors such as inadequate feeding frequency, types of complementary foods, breastfeeding duration, and low family income. These findings highlight the focus and potential direction for early identification and intervention.

## Introduction

Early childhood is a crucial period for children's cognitive, language, social, emotional, and motor development as the brain develops rapidly in the early years, especially during the first 1,000 days. It is a period not only of great opportunity for all-around learning but also of vulnerability to the negative impacts from the surrounding environment. For instance, the neuroplasticity during this period makes the child particularly vulnerable to external environmental stimulation such as nutritional status, socioeconomic status, and the parenting environment (1, 2). Early childhood developmental conditions often affect children throughout their lifespan and manifest in various areas (3). Some previous evidence had demonstrated that children with developmental delay are at higher risk for poor academic performance, career development, and mental health disorders in the future than those without delay (4–6). Although early childhood development has begun to receive increasing attention globally, it was estimated that there are still 249 million children under the age of 5 in low- and middle-income countries at risk of developmental delay, and approximately 7% (17 million) of these children are from China (2). However, few studies have investigated the status of developmental delay, especially in poor rural China (7).

The nutritional status of children and feeding practices are important factors in meeting basic physiological needs of children in early childhood development. Infancy is a period of rapid transition from a diet of nothing but milk to a varied diet of nearly all foods for most infants. An unscientific feeding diet and nutrition in this period can lead to deficiencies in essential micronutrients, which may affect the immune system's function and have lasting effects on children's growth and development (8, 9). For example, in the early years, iron deficiency and anemia are significant risk factors for physical, cognitive, and mental development in children (10, 11). The period of 0–2 years are the most sensitive period for stunting (12, 13), and the nutritional status during this period was significantly associated with later cognition, executive function, and school performance, but this association is not as strong after 24 months (14).

Children in poor rural areas of China face certain disadvantages in terms of nutritional status. First, nutrition knowledge and infant feeding practices were poor in rural China. In addition, according to National Statistics Bureau data, there were 6.97 million left-behind children in rural areas since their parents migrated for work in urban areas, children under 5 years accounted for 21.7% of the left-behind children, and 96% of the left-behind children were cared for by their grandparents (15). Previous studies have found that grandparents pay less attention to the nutritional quality of children's diets (16). A study of complementary feeding practices in rural China revealed a severe lack of diversity in children's diets, especially iron-rich foods such as meat (17). There has been a fair amount of research on what a child should be fed during the transitional period (18–21) but little on the correlation between feeding practices and early childhood development. Moreover, studies on children's feeding behavior during the transition phase in rural China are still scarce.

The objective of this study was to investigate the association between the early development status of children and feeding practices during the transition period in poor rural areas of China to provide suggestions for future interventions in the early development of children. First, we aimed to describe the early developmental status of children aged 6–23 months in poor rural areas of China. Second, we compared the feeding practices of caregivers to study their differing understanding of nutritional needs when they were raising children with different characteristics. Finally, we examined the correlation between child feeding practices and early childhood development and explored how the factors of children's nutrition status and caregivers' feeding practices affect the developmental delay of children.

## Methods

### Sample and data collection

The survey was conducted as part of the Integrated Early Child Development (IECD) Project, a community-based cross-sectional survey focusing on the health, nutrition, and developmental status of infants and young children in 83 villages across Guangxi and Shaanxi provinces. All villages participating in the survey met the following inclusion criteria: (1) more than 50 children over the age of 3 years, as well as their caretakers, were eligible to participate in the survey and (2) the village had at least one community health service center with professionals that could help with the investigation.

All children aged 1–35 months in these villages were eligible for recruitment. The survey reached 2,953 of 4,288 children, and the response rate was 68.9%. We excluded 16 children with language impairments or disabilities and 100 caregivers who were not the child's primary caregiver (i.e., the person who took care of the child most of the time in the family) because only the primary caregiver could accurately recall the child's feeding practices. To ensure an adequate sample size, we used the traditional cross-sectional random sampling formula, *n* = Z^2^_a_P (1 – P)/*d*^2^. Based on a study conducted in Brazil, the estimated prevalence of early childhood development delays was ~21.4% (22). Controlling for a margin of error of 0.1P, the sample size needed to be no < 1,600 children. In our study, we specifically selected children aged 6–23 months and finally included 1,631 children for analysis, fulfilling the previously estimated sample size requirement.

### Measurement

The measurement of data relied primarily on questionnaires and related scales. The questionnaire was based on UNICEF's fifth Multiple Indicator Cluster Survey (MICS5) (23), and it included basic information concerning the child, their caregiver, and their family. The feeding practices of children, including whether they were bottle-fed, the duration of breastfeeding, the frequency of feeding, and the types of complementary foods consumed in the past 24 h, were also investigated.

#### Ages and Stages Questionnaire

The prevalence of neurodevelopmental delay, our outcome indicator, was assessed using the Ages and Stages Questionnaire (ASQ) (third edition, Chinese version) (24). This questionnaire is a brief, standardized screening tool used to identify potential developmental delays in five domains: communication, gross motor skills, fine motor skills, problem-solving, and personal-social skills. Each domain consists of six questions, and if a child scores below the threshold in any domain, it suggests a potential developmental delay.

#### Infant and child feeding index

To comprehensively evaluate feeding practices in infants and young children, we utilized the infant and child feeding index (ICFI). This index is a set of simple, valid, and reliable indicators based on the method proposed by Ruel and Menon (25), the feeding recommendations of the World Health Organization (WHO) (26), and the ICFI scoring system developed by the Nutrition and Health Survey of the Chinese People (27). It has been widely used in previous studies to assess feeding practices for children aged 6–23 months (28, 29). The ICFI covers four key dimensions of child feeding: breastfeeding, bottle feeding, frequency of feeding, and types of complementary foods. In Table 1, we provide an explanation of the variables and scoring system based on the ICFI.

**Table 1:** Score of variables regarding feeding habits in ICFI.

**Indicator name**	**6–8 months**	**9–23 months**
**Breastfeeding**	Yes = +2	Yes = +2
	No = 0	No = 0
**Bottle feeding**	Yes = 0	Yes = 0
	No = +1	No = +1
**Frequency of complementary foods**		
Breastfed children	0 times = 0	0 times = 0
	1 times = +1	1–2 times = +1
	≥2 times = +2	≥3 times = +2
Non-breastfed children	0–1 times = 0	0–2 times = 0
	2–3 times = +1	3–4 times = +1
	≥4 times = +2	≥5 times = +2
**Types of complementary food**	0–1 kinds = 0	0–1 kinds = 0
	2–3 kinds = +1	2–3 kinds = +1
	≥4 kinds = +2	≥4 kinds = +2
**Total score**	7 points	7 points

#### Dietary variety

To describe the variety of foods in infants' diets, we categorized the foods into eight groups based on their regular consumption: (1) breastmilk, (2) grains, roots, and tubers, (3) legumes and nuts, (4) dairy products (milk, infant formula, yogurt, and cheese), (5) flesh foods (meat, fish, poultry, and liver/organ meats), (6) eggs, (7) vitamin A-rich fruits and vegetables, and (8) other fruits and vegetables (30). Caregivers were asked to recall which food groups the children had consumed in the past 24 h, and if a child had eaten any food from a particular group, then it was considered as having consumed from that particular food group. According to WHO recommendations, if the types of complementary food reached five of the eight food groups, we considered it qualified.

#### Feeding frequency

The percentage of children who were fed at a frequency of minimum number of times or more in the past day was used to evaluate the percentage of children who met the qualified feeding frequency requirements. As this indicator is designed to evaluate energy intake from foods other than breast milk, it differs for breastfeeding and non-breastfeeding children. According to the definition of the ICFI, the specific feeding requirements for breastfeeding and non-breastfeeding children are as follows: (1) breastfeeding children: solid, semi-solid, or soft foods, two times for infants aged 6–8 months and three times for children 9–23 months; (2) non-breastfeeding children: solid, semi-solid, or soft foods or milk feeds, four times for children aged 6–23 months (30).

### Statistical analysis

The developmental delay status of children was described by frequency. Multivariate logistic regression analyses were applied to examine the influencing factors of children's psychological behavior development. Correlation analysis was conducted first; then, we incorporated all the significant factors (*p*-value < 0.05) in the univariate analysis into the multiple linear regression model. In addition, the child's gender, month age, ethnicity, whether left-behind children, caregiver type and education level, and family income were regarded as the covariates of influencing factors on feeding methods. Logistic regression analyses were also applied to identify the specific factors influencing the development of different functional areas of developmental delay in children. The statistical significance was defined as a *P*-value of < 0.05. All data analyses were performed using SPSS 22.0.

## Result

### Study population

As shown in Table 2, among the children, 56.6% were boys, and children of Han nationality accounted for 65.0% of the study population. No significant differences in children's age and gender distribution were observed. The mean age of the caregivers was 29.7 ± 9.7 years. Of these, 79.9% were mothers, 7.6% were fathers, and 12.5% were other relatives. A total of 66.2% of caregivers had a junior high school diploma or higher.

**Table 2:** Characteristics of the children, caregiver, and family (*N* = 1,631).

**Characteristic of children**	***N***	**Distribution**	**Characteristic of caregivers**	***N***	**Distribution**
**Gender**			**Primary caregivers**		
Boys	923	56.6	Mother	1,303	79.9
Girls	708	43.4	Father	124	7.6
**Ethnicity**			Non-parent	204	12.5
Ethnic han	1,060	65.0	**Age of caregivers**		
Ethnic minority	571	35.0	< 20	36	2.2
**Age group (months)**			20~	1,045	64.1
6–11	542	33.2	30~	323	19.8
12–17	494	30.3	40~	92	5.6
18–23	595	36.5	50~	120	7.4
**Left-behind children**			**Caregivers' education**		
Yes	892	54.7	Illiteracy	154	9.4
No	739	45.3	Primary school	397	24.3
			Junior high school	853	52.3
			High school and above	227	13.9
			**Family income** ^*^		
			≤ 2,300	261	20.3
			>2,300	1,022	79.7

^*^Annual household income per capita of RMB¥ 2,300 is the poverty threshold in rural China in 2013.

### Suspected developmental delay among children aged 6–23 months

Overall, 33.6% of the surveyed children aged 6–23 months had suspected developmental delay in at least one region. The prevalence of suspected developmental delay tends to decrease as the age of the child increases. The lowest prevalence of gross motor skill delays (8.9%) and the highest prevalence of fine motor skill delays (17.7%) were found in all domains; the prevalence in other domains ranged from 11.9 to 14.9%. In addition, the primary caregivers and family income were also the factors that affected the children's overall developmental delay and most of the domains of developmental delay (Table 3).

**Table 3:** Univariate analysis of factors related to developmental delays in five domains and overall ASQ (comparison with the chi-squared test).

**Domain**	**ASQ overall**	**Communication**	**Gross motor**	**Fine motor**	**Problem-solving**	**Personal-social**
**Gender**						
Boys	35.5	15.3	10.7	18.0	13.7	11.1
Girls	31.2	14.5	13.2	17.3	14.0	12.9
**Month age**						
6–11	35.4	19.7^*^	9.2	20.6	11.4^**^	10.3^*^
12–17	33.5	14.6	9.3	18.2	17.6	15.0
18–23	32.0	10.8	16.3^**^	14.7	12.9^*^	10.7^*^
**Caregivers' education**						
Junior high school and below	34.8^**^	15.4	12.1	18.5^*^	14.8^**^	12.3
High school and above	26.1	11.9	10.9	12.7	7.5	9.3
**Primary caregivers**						
Mother	30.5	13.3	10.6	15.8	11.4	10.9
Father	49.6^**^	21.8^**^	16.4	23.1^*^	20.5^**^	13.0
Non-parent	43.5^**^	21.2^**^	16.7^*^	26.^6**^	25.3^**^	17.2^**^
**Family income**						
≤ 2,300 (poverty)	25.8^**^	19.7^**^	19.0^**^	24.5^**^	21.3^**^	17.4^**^
>2,300	17.9	13.2	8.8	14.1	10.6	9.9
Overall	33.6	14.9	11.8	17.7	13.8	11.9

PS: ^*^represents *P* ≤ 0.05, ^**^represents *P* ≤ 0.01.

### Feeding practice

**Figure 1:**
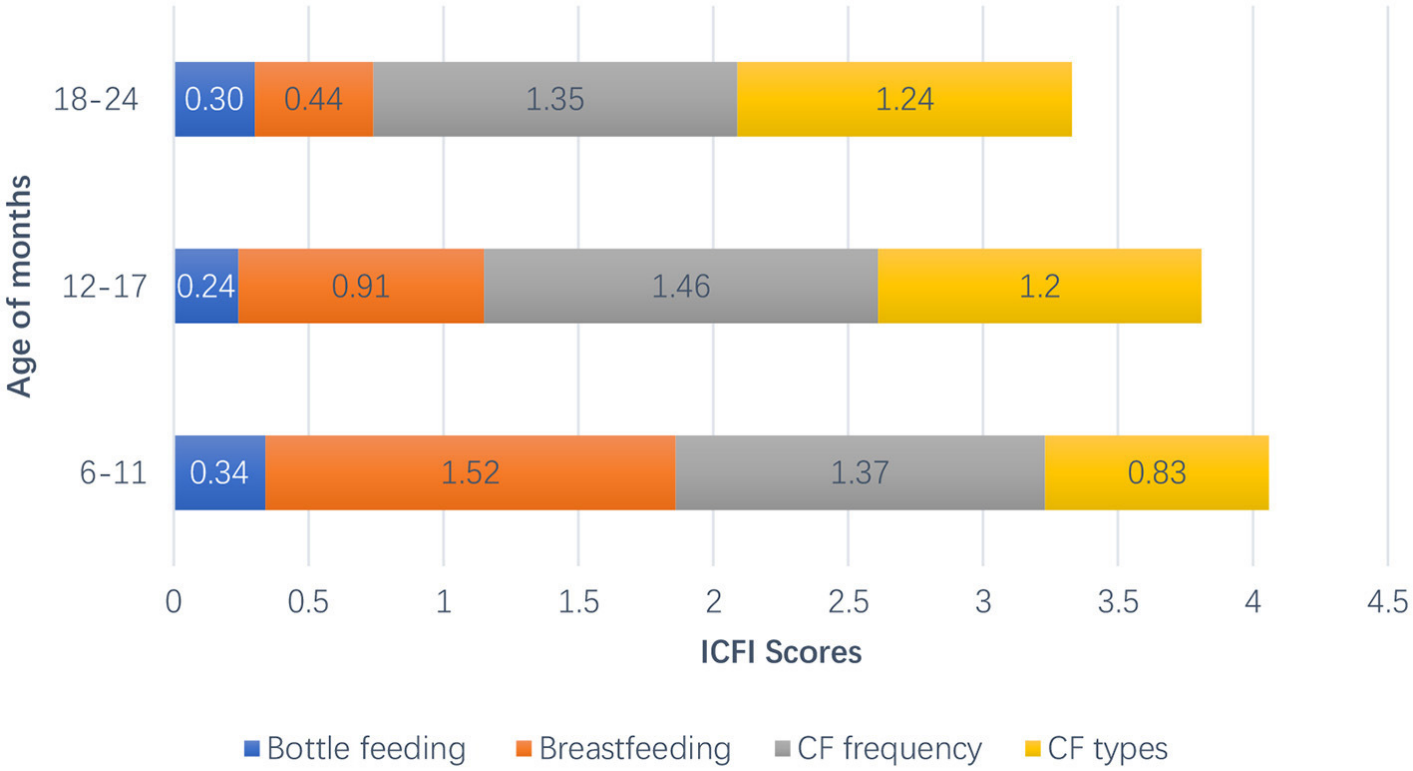
The trend of ICFI with child's age of months.

The proportion of infants under the age of 2 who have never been breastfed reached 11.7%. A total of 46.8% of mothers stopped breastfeeding, and the mean weaning age was 10 ± 4.32 months. Although 55.4% of children meet the requirements of supplementary food types, only 32.6% meet the frequency requirements (Table 4). The mean of the ICFI of children aged 6–23 months was 3.79 ± 1.58. The ICFI of children whose caregivers were mothers or fathers was significantly higher than that of children with non-parent caregivers. The ICFI was higher in children from higher-income families and when their caregiver had a higher level of education. In addition, the ICFI score decreased significantly as children's months of age increased. The main reason for this was the significant decrease in breastfeeding scores (Figure 1).

**Table 4:** Status of feeding practices of children aged 6–23 months.

	**Means of ICFI**	***P***
**Gender**		0.102
Boys	3.90 ± 1.54	
Girls	3.64 ± 1.61	
**Left-behind children**		0.263
Yes	3.85 ± 1.56	
No	3.72 ± 1.59	
**Primary caregivers**		**< 0.001**
Mother	3.92 ± 1.57	
Father	3.63 ± 1.59	
Non-parent	2.93 ± 1.31	
**Caregivers' education**		**0.019**
Junior high school and below	3.75 ± 1.59	
High school and above	4.04 ± 1.44	
**Family income**		**0.009**
≤ 2,300 (poverty)	3.53 ± 1.62	
>2,300	3.93 ± 1.54	

### Influencing factors of children's developmental delay in feeding practices

**Figure 2:**
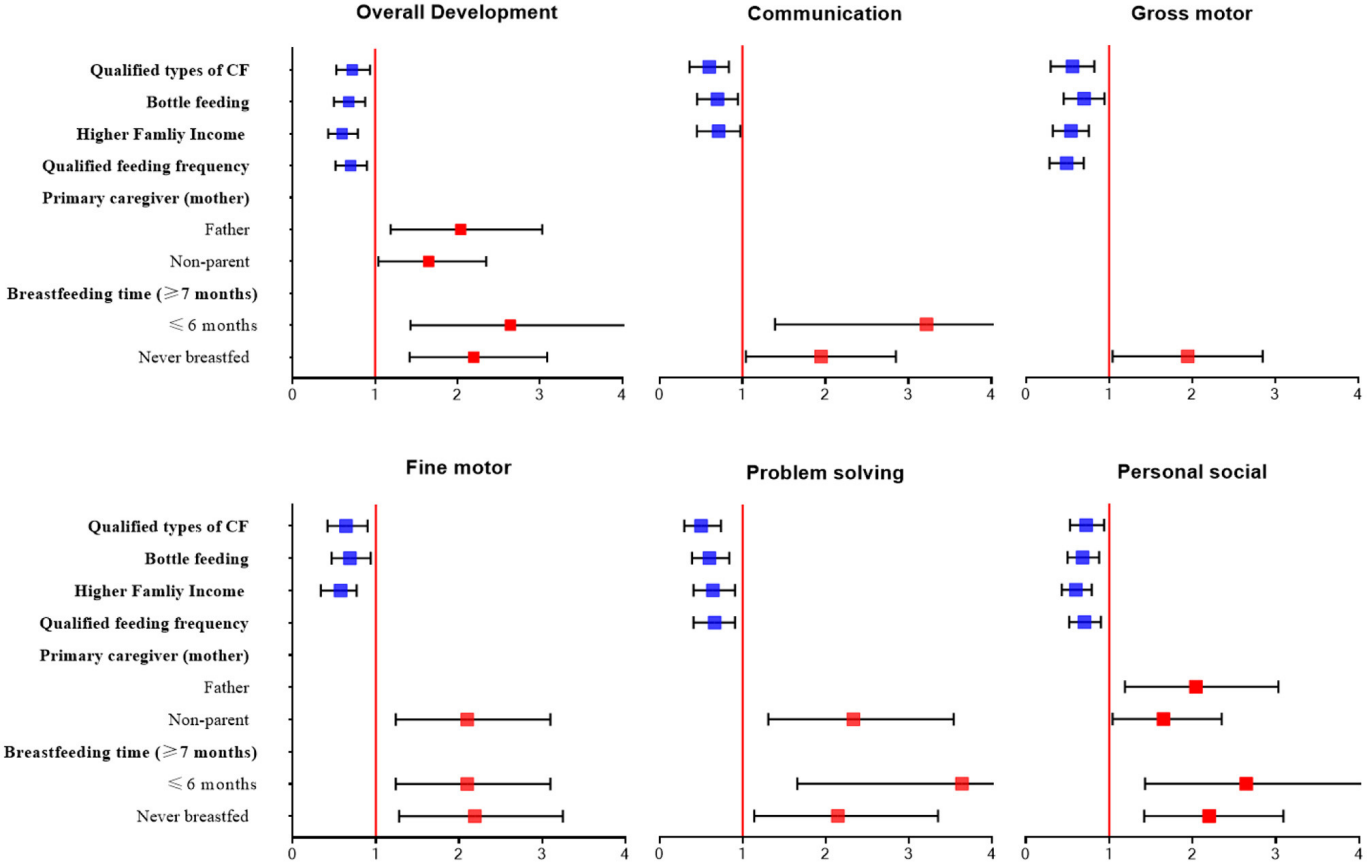
Multiple logistic regression analysis for exploring factors related to developmental delay.

Based on the possible factors related to early childhood development found in univariate analysis, we incorporated all significant factors as the independent variables into the multiple regression model. Figure 2 shows the results of the multivariate logistic regression analysis of the feeding practice factors that influence developmental delay among all children. First, bottle feeding (OR = 0.66, 95% CI: 0.50–0.88) was determined as the protective factor of early development. Furthermore, children who had a wider variety of complementary foods and an adequate feeding frequency were significantly more likely to have better developmental results compared to those who did not (OR = 0.70, 95% CI: 0.53–0.94; OR = 0.69, 95% CI: 0.52–0.90). Children in a family with an income higher than RMB¥ 2,300 were significantly less likely to be stunted than those with an income of RMB¥ 2,300 and lower. In addition, we found that both never breastfeeding and breastfeeding < 6 months were risk factors for early childhood development (OR = 2.09, 95% CI: 1.42–3.09; OR = 2.41, 95% CI: 1.43–4.09).

## Discussion

### Prevalence of developmental delay and feeding status

With the social and economic development in rural areas of China, there has been a continuous decrease in infant and child mortality rates. However, the developmental status of children under the age of 5 years has received little attention, and the majority of them receive insufficient support. According to the 2010 Chinese population census, 4.5 million children under the age of 3 in poor areas of China might have developmental delays (31). Our study specifically focused on children aged 6–23 months and found that the prevalence of suspected developmental delays ranged from 10 to 20% in different regions, with fine motor delays being the most prevalent at 17%. The WHO has reported limited data on developmental difficulties in early childhood within low- and middle-income countries, citing prevalence rates of 17% in Senegal, 15% in Nigeria, 13% in India, and 24% in Brazil (32). The variations in rates of developmental difficulties found in different countries can be attributed to differences in how developmental delay is defined. Given the high sensitivity of the ASQ used in our survey, the results yielded comparatively higher prevalence rates than those reported by the WHO for other countries. The lower prevalence rates reported in other developing countries may indicate an underestimation or imprecise measurement of developmental delay. In conclusion, ECD in poor rural areas of China requires further attention, with a special focus on the development of fine motor skills.

We found no significant differences between boys and girls, either in feeding practices or developmental delays. This contrasts with the traditional Chinese custom of prioritizing male children over female children in rural areas (33). It suggests that this phenomenon may be improving in poor rural areas of China. Socioeconomic conditions of the family were significant influencing factors on ECD (34, 35). Recent research in neuroscience has shown a correlation between family poverty levels in a child's first 3 years of life and the gray matter volume in their brains (36), which has been linked to worse cognitive performance and reduced cortical brain volumes in future (37). Combining child nutrition and early development interventions has been found to effectively mitigate the effects of early childhood adversity caused by poverty and provide opportunities for nutritional supplementation and early learning (38).

### Breastfeeding practices and ECD

According to guidelines from the WHO and UNICEF, infants should be exclusively breastfed for the first 6 months after birth (39, 40). However, our survey found that more than 10% of children aged 6–23 months had never been breastfed, and nearly half had discontinued breastfeeding. Previous research has focused on the association between breastfeeding and children's intelligence or physical development (41). The well-known nutritional benefits of breastfeeding include the presence of over 200 unique types of human milk oligosaccharides (HMOs), which promote the growth of beneficial bacteria and effectively prevent diarrhea and infant mortality (42, 43). The presence of long-chain polyunsaturated fatty acids in breast milk also contributes to the development of infants' intelligence (44). Additionally, breastfeeding appears to affect other areas of early development, such as communication and social skills. This suggests that breastfeeding not only provides nutrients for the infant but also creates an environment that stimulates the child's cognitive and learning abilities (45). Furthermore, breastfeeding behavior, particularly its duration, promotes parent–child attachment, which may affect the child's overall health and ability to cope with stressful situations (41).

An unexpected finding from our study was that bottle feeding also emerged as a protective factor for ECD. Bottle feeding, also known as “breastmilk fed by bottles”, is a common choice of feeding behavior in modern society (46). However, due to the unhygienic practices associated with bottle feeding, it can increase the risk of diarrhea symptoms in children (47). Possible reasons for this phenomenon could be that caregivers who use bottle feeding may possess more knowledge than those who exclusively breastfeed about the available and consumed quantity of milk, which may influence feeding decisions based on bottle-based cues (48). Additionally, a study found that the average amount of milk consumed by bottle-fed or mixed-fed infants was significantly higher than that consumed by breastfed infants (49). It remains unclear whether bottle feeding directly affects child development and what the underlying mechanisms might be. Further research is needed to confirm this finding.

### Nutritional supplements and ECD

Scientific complementary feeding, in addition to breastfeeding, was found to be another protective factor for ensuring nutritional quality and child development. While there is substantial literature on the importance of breastfeeding or formula feeding for ECD, there are fewer publications and less evidence focusing on the addition of complementary foods. Eating behaviors established in childhood can have a lasting impact on a child's long-term diet and overall health (50). However, most stunting in children occurs within the first 2 years after birth when they have high nutritional needs. Ensuring the quality and quantity of their diets during this time, particularly after the period of exclusive breastfeeding, can be challenging (51). Therefore, nutritional supplementation before the age of 2 years seems to have a more significant and long-term impact on children's future development. Previous studies have found that macronutrient supplementation significantly affects intellectual development in children up to 24 months (52). Furthermore, early childhood nutritional quality has a significant impact on future wage levels, but this effect diminishes after 36 months (53).

The results of this study also demonstrate a significant association between dietary diversity and ECD. The minimum dietary diversity for all infants in our survey was low, at 32.6% (reaching five out of eight food groups), with the majority of supplemented food being starch-based staple food. Although children had a certain intake of vegetables and fruits, there was a lack of high-quality vegetables or fruits rich in vitamin A. Although children consumed some vegetables and fruits, there was a noticeable lack of high-quality vegetables or fruits rich in vitamin A. The proportion of protein-rich meat food supplements was also relatively low. Rural economic conditions limited caregivers' purchasing ability and willingness. A large number of left-behind children in China's rural areas depend on their family's economic status and the village where they live for nutritional supplementation as their parents who work away from home cannot directly provide food for them. These results do not meet the dietary diversity needs of children. There are two main aspects to the mechanism by which complementary feeding influences ECD. First, certain nutrients in complementary foods, such as long-chain polyunsaturated fatty acids (LCPUFAs) and docosahexaenoic acid (DHA), may alter cortical function maturation of the brain and play an important role in brain development (54, 55). Second, neuropsychological studies have shown that rapid growth in early childhood makes the brain particularly susceptible to stimulation by the external environment. Different types of complementary foods can provide children with more gustatory and tactile stimulation, which in turn can influence their development (56).

During the complementary feeding period, feeding frequency is an important determinant of nutritional supplementation in children. Previous studies have found that complementary feeding frequency is associated with stunting but not wasting or underweight (57, 58). Combined with this study's results, it is suggested that adequate feeding frequency is equally important for children's development. While the frequency of complementary feeding for children aged 6–23 months is essential for various nutritional supplements, no clear evidence of a causal association between feeding frequency and ECD has been found in other studies. We speculate that feeding may be a cognitively stimulating behavior for children, creating more opportunities for interaction between the child and caregiver during the process. This interaction, such as reactive feeding, can directly affect food acceptance, dietary intake, and overall development status (59).

### Recommendations

We make the following recommendations based on our findings. First, the results demonstrated a significant correlation between unscientific feeding practices during the transition from breastfeeding to complementary feeding and early childhood development. This is a promising finding that could highlight the need for future research projects in family parenting environments and feeding practice interventions, especially in poor rural areas. Second, ensuring a balanced and nutritious diet for young children is crucial for their optimal growth and development. It is recommended that a child's diet should be diverse, incorporating a combination of different food groups. This includes fruits and vegetables rich in vitamins and minerals, protein sources such as meat, fish, eggs, or legumes, as well as whole grains for energy and fiber. Furthermore, breastfeeding plays a crucial role in early childhood nutrition. According to WHO recommendations, children should be breastfed during the first 6 months of their lives. In conclusion, early childhood feeding behaviors, including a nutritious diet and encouraging breastfeeding, are fundamental to a child's health and development. It is important that caregivers are educated and supported in these practices to ensure the best possible start in life for their children.

### Strengths and limitations

This study has several strengths, including a large sample size and a comprehensive evaluation of development status among children in poor rural areas of China. Additionally, there is a relative lack of research on the transition from exclusive to mixed breastfeeding at 6–23 months of age in the field of ECD, and this study provides data support and future research directions.

However, this study also has some limitations. First, it investigated children's feeding practices without measuring the feeding interaction between caregiver and child. Second, the study's indicators were based on a self-reported 24-h recall survey, rather than a long-term review of children's diets. Third, the measurement of feeding practices was insufficient, resulting in a weak interpretation of the results. The study focused only on breastfeeding duration, which has significant limitations. The mechanisms of influence through which breastfeeding behavior affects ECD via nutritional supplementation and the nurturing environment are highly heterogeneous. Although we have suggested several possible influence mechanisms, they need further verification in future studies. Additionally, this study only revealed correlations between various feeding behavior factors and ECD, without measuring potential mediators or moderators in the pathway of feeding behavior to ECD. Therefore, we can only propose hypotheses about these potential influence mechanisms and provide references for further longitudinal research in future.

## Conclusion

In conclusion, a high prevalence of developmental delay was observed in children during the dietary transitional period in rural areas of China. The feeding practices of caregivers in this area also exhibited certain problems, such as dietary diversity or breastfeeding behavior. This survey explored various influencing factors significantly associated with ECD status, including inadequate feeding frequency and types of complementary foods, breastfeeding duration, and low family income. These factors highlight the focus and potential direction for early identification and intervention. An in-depth, multi-domain understanding of feeding practices during the dietary transitional period is necessary to promote the future development of children.

## Data availability statement

The raw data supporting the conclusions of this article will be made available by the authors, without undue reservation.

## Ethics statement

The studies involving human participants were reviewed and approved by Peking University's Biomedical Ethics Review Committee. Written informed consent to participate in this study was provided by the participants' legal guardian/next of kin.

## Author contributions

YL completed the first draft and conducted statistical analyses. CC managed the manuscript. Both authors have approved the final manuscript for submission.
